# Significance of *microRNA-related* variants in susceptibility to recurrence of oropharyngeal cancer patients after definitive radiotherapy

**DOI:** 10.18632/oncotarget.9014

**Published:** 2016-04-26

**Authors:** Xingming Chen, Erich M. Sturgis, Chengyuan Wang, Xiaoli Cao, Yuncheng Li, Qingyi Wei, Guojun Li

**Affiliations:** ^1^ Department of Otolaryngology-Head and Neck Surgery, Peking Union Medical College Hospital, Peking Union Medical College and Chinese Academy of Medical Sciences, Beijing 100730, China; ^2^ Department of Head and Neck Surgery, The University of Texas MD Anderson Cancer Center, Houston, TX 77030, USA; ^3^ Department of Epidemiology, The University of Texas MD Anderson Cancer Center, Houston, TX 77030, USA; ^4^ Department of Otolaryngology-Head and Neck Surgery, China-Japan Friendship Hospital, Beijing 100029, China; ^5^ Department of Ultrasound, Yantai Yuhuangding Hospital, Yantai, 264000, China; ^6^ Department of Otolaryngology, Union Hospital of Tongji Medical College, Huazhong University of Science and Technology, Wuhan, 430022, China; ^7^ Duke Cancer Institute, Duke University Medical Center, Durham, NC 27710, USA

**Keywords:** miRNA, recurrence, oropharyngeal cancer, HPV, variants

## Abstract

Common single nucleotide polymorphisms (SNPs) in *miRNAs* may affect miRNA functions and their target expression and thus may affect biological activities and cancer etiology as well as prognosis. Thus, we determined whether the 9 SNPs in *microRNAs* modify the risk of recurrence of squamous cell carcinoma of the oropharynx (SCCOP) in a cohort of 1008 patients. The log-rank test and multivariate Cox models were used to evaluate the associations. We found that the SNPs in the miRNA146, miRNA196, and Gemin3 were associated with a significantly reduced and increased risk of SCCOP recurrence after multivariate adjustment (aHR, 0.6, 95%CI, 0.4-0.9, aHR, 2.1, 95%CI, 1.6-2.8, and aHR, 0.6, 95%CI, 0.5-0.9, respectively). Furthermore, the similar effect of these 3 SNPs on SCCOP recurrence risk was found in HPV-positive SCCOP patients only. However, no significant associations were found for other SNPs. To evaluate the aggregate effects of these SNPs, we performed a combined risk genotype analysis. We found that, compared with the low-risk reference group with less than 4 risk genotypes, the medium-risk group with 4 or 5 risk genotypes exhibited a 1.7-fold (1.2-2.4) increased risk whereas the high-risk group with more than 5 risk genotypes exhibited a 3.0-fold (1.7-4.2) increased risk (P_trend_ < 0.001). Such combined effects were particularly pronounced in HPV-positive SCCOP patients. Taken together, this is the first study with a large cohort of SCCOP patients showing that *miRNA*-related genetic variants may modify risk of SCCOP recurrence individually and jointly. Larger studies are needed to validate these results.

## INTRODUCTION

Despite the decrease in smoking rate, the incidence of SCCOP has increased rapidly in recent years because of the increasing infection of human papillomavirus (HPV) [1–4]. Several modalities for SCCOP treatment are successful, while recurrence for SCCOP remains a major clinical problem for prognosis due to a high rate of recurrence [5]. Even the progress in therapeutic strategies and approaches for SCCOP, the patients with treatment and disease recurrence have a very low 5-year survival [6]. A new biomarkers, which may more accurately predict recurrence in SCCOP patients would help identify patients at high-risk for recurrence. There are many risk factors for SCCOP recurrence, while not every patient after treatment develops disease recurrence. We might conclude that other unknown risk factors, such as host genetic factors, affect the risk of SCCOP recurrence.

MicroRNAs (miRNAs) play critical roles in a broad range of biological activities [7–9]. Studies have shown that miRNAs may not only affect cancer development, early detection, diagnosis and prognosis [9–13] but also the regulation of immune/inflammation or cell death responses [14, 15]. Genetic polymorphisms of *miRNAs* may modify response to cytotoxic therapy for SCCOP through both of these response pathways. After the transportation from nucleus to cytoplasm, pre-miRNAs are developed to the mature miRNAs by a protein complex which includes TRBP, Gemin4, and Gemin3. The produced miRNAs can affect many gene expressions when they bind to the 3′ untranslated region (UTR) of their target genes. Genetic variants of *miRNAs* (e.g., single nucleotide polymorphisms: SNPs), could result in functional changes of their host miRNAs, leading to different expressions of their target genes. Such changes thus may affect gene functions.

Previous studies have reported that several SNPs, such as *pre-mir-146a, hsa-mir-196a2* C>T, and *hsa-mir-499* A>G, may result in different miRNA expressions and affect cancer risk and survival [16, 17]. However, as yet, no study has simultaneously investigated whether *miRNA* SNPs affect risk of recurrence among SCCOP patients. In current study we selected a total of 9 SNPs for study, including 6 SNPs in pre-*miRNAs* and 3 promoter SNPs in *miRNA* biogenesis genes (MAF > 0.05) [18]. We performed the genotyping for 9 SNPs to test our hypothesis that combined *miRNA*-related SNPs modulate treatment responses and prognosis. In the end, we assessed the association between the *miRNA*-related variants and risk of recurrence, and such analysis was further stratified by patient's tumor HPV16 status among this large incident SCCOP cohort.

## RESULTS

In this study, as shown in Table 1, a total of 1008 patients were included for our final analysis. The median follow-up time and mean age at diagnosis among overall patients, recurrent patients, and patients without recurrence were previously reported [19]. The SCCOP recurrence types, 5-year recurrence rate, and the associations of those variables, including sex, age, ethnicity, smoking, alcohol use, comorbidity, index cancer stage, and treatment with DFS were also previously reported [19].

**Table 1:** Characteristics of patients with SCCOP (N = 1008)

Variable	No. (%) of patients	No. of patients with recurrence	5-year recurrence rate (%)	*P*^a^ value
No. of patients	1008 (100)	181	0.20	
Age				
≤ 57 years	621 (61.6)	85	0.15	< 0.0001
> 57 years	387 (38.4)	96	0.27	
Sex				
Male	872 (86.5)	161	0.20	0.3110
Female	136 (13.5)	20	0.19	
Ethnicity				
Non-Hispanic white	913 (90.6)	146	0.17	< 0.0001
Other	95 (9.4)	35	0.41	
Smoking				
Never	388 (38.5)	51	0.14	0.0004
Ever	620 (61.5)	130	0.23	
Alcohol use				
Never	247 (24.5)	26	0.10	0.0005
Ever	761 (75.5)	155	0.23	
Comorbidity				
None or mild	913 (90.6)	157	0.19	0.0370
Moderate to severe	95 (9.4)	24	0.27	
Index cancer stage				
1 or 2	72 (7.1)	11	0.19	0.5280
3 or 4	936 (92.9)	170	0.20	
Treatment				
X/XC/XS/S	947 (93.9)	166	0.19	0.0030
SXC	61 (6.1)	15	0.32	

a *P*: Log-rank test for DFS between the two groups. X, radiotherapy; C, chemotherapy; and S, surgery.

**Figure 1A-B:**
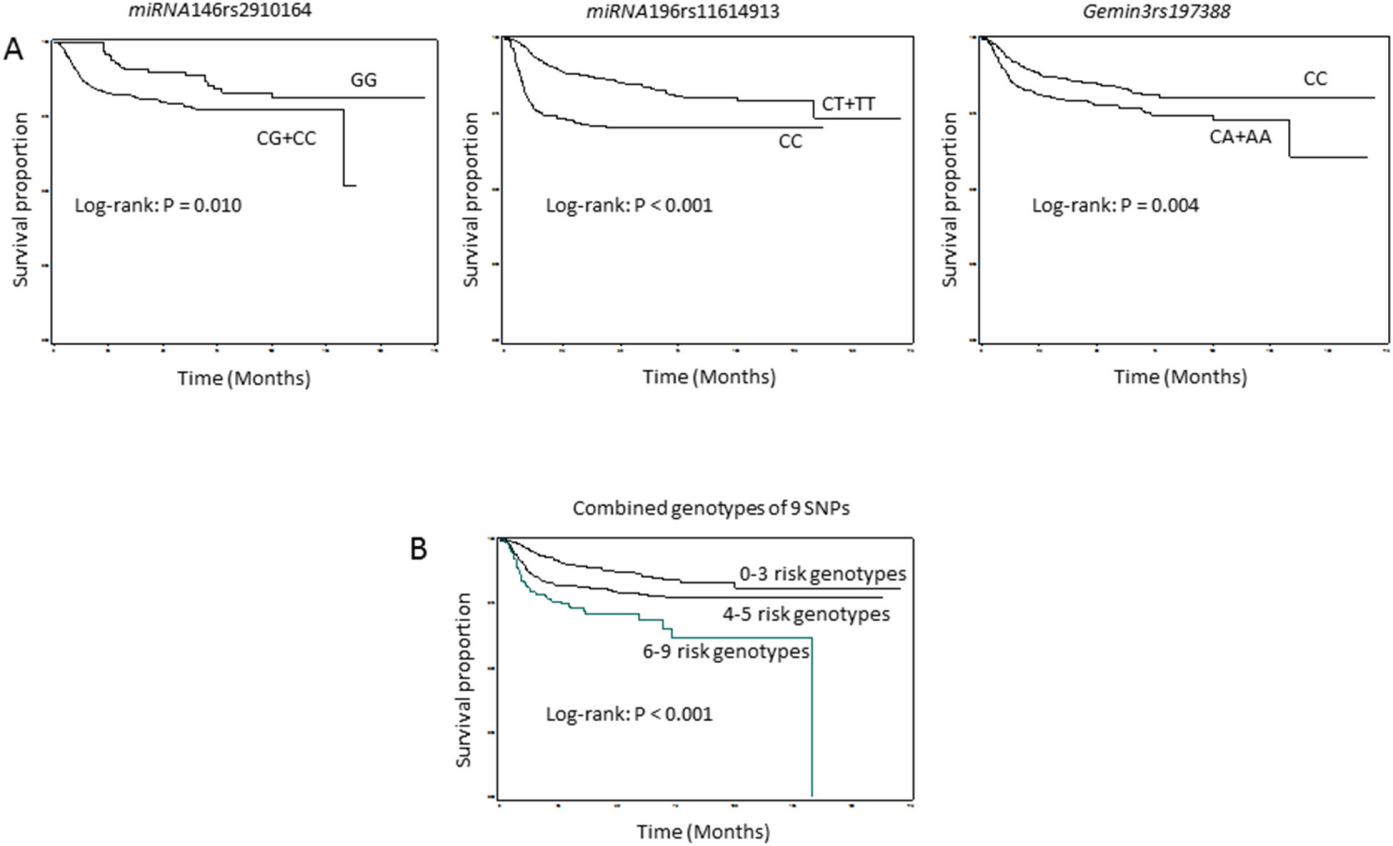
Kaplan-Meier estimates of the cumulative recurrence rates in patients by *miRNA*-related genotypes (N = 1008)

The SCCOP patients with the *miRNA146*rs2910164 and Gemin3rs197388 common homozygous genotypes had significantly better DFS than those with the corresponding variant genotypes, respectively (*P_Log-rank_* = 0.010 and *P_Log-rank_* = 0.004), while patients with *miRNA196*rs11614913 common homozygous genotype had significantly worse DFS than those with the variant genotypes, respectively (*P_Log-rank_* < 0.0001) (Figure 1A). We did not observed significant differences in DFS between different genotypes of other 6 polymorphism (All *P_Log-rank_* > 0.05). To evaluate the combined associations for the 9 polymorphism, a multivariable analysis was conducted. This multivariable Cox models included the adjusted variables such as age, sex, ethnicity, smoking status, alcohol status, comorbidity, stage, and treatment (Table 2). We found that patients carrying *miRNA146*rs2910164 GG and Gemin3rs197388 CC genotypes had an approximately 40% significantly lower risk of recurrence than those carrying *miRNA146*rs2910164 CG+CC and Gemin3rs197388 CA+AA genotypes, (aHR, 0.6; 95% CI, 0.4-0.9; and aHR, 0.6; 95% CI, 0.5-0.9, respectively). However, the patients with *miRNA196*rs11614913 CC genotype had an approximately 2 times significantly increased recurrence risk (aHR, 2.1; 95% CI, 1.6-2.8) than those carrying *miRNA196*rs11614913 CT+TT genotypes.

**Table 2:** Association between *miRNA-*related genotypes and SCCOP recurrence in patients with SCCOP (N = 1008)

Genotype	No. of rec. of patients/no. of patients	5-year rec. rate	Log-rank *P*	aHR*, 95% CI
*miRNA146rs2910164*				
GG (ref.)	20/152	0.10	0.010	0.6 (0.4-0.9)
CG+CC	161/856	0.20		1.0
*MiRNA196rs11614913*				
CC (ref.)	72/261	0.30	< 0.001	2.1 (1.6-2.8)
CT+TT	109/747	0.15		1.0
*MiRNA149* rs2292832				
CC (ref.)	89/490	0.19	0.852	1.0 (0.7-1.3)
CT+TT	92/518	0.18		1.0
*MiRNA499* rs3746444				
TT (ref.)	116/582	0.20	0.100	1.4 (0.9-1.7)
CT+CC	65/426	0.16		1.0
*MiRNA492rs2289030*				
CC(ref.)	147/829	0.18	0.389	0.9 (0.6-1.3)
CG+GG	34/179	0.20		1.0
*MiRNA423rs6505162*				
TT(ref.)	22/119	0.20	0.759	1.1 (0.7-1.8)
CT+CC	159/889	0.18		1.0
*Gemin4rs910924*				
CC(ref.)	60/385	0.16	0.077	0.8 (0.6-1.1)
CT+TT	121/623	0.20		1.0
*Gemin3rs197388*				
CC(ref.)	80/537	0.15	0.004	0.6 (0.5-0.9)
CA+AA	101/471	0.22		1.0
*TRBPrs784567*				
CC(ref.)	69/362	0.19	0.579	1.2 (0.9-1.6)
CT+TT	112/646	0.18		1.0

HR, hazard ratio.

* Adjusted for age, sex, ethnicity, smoking status, alcohol use status, stage, comorbidity, and treatment.

The effects of 9 SNPs on risk of recurrence were then assessed among 324 HPV16-positive [HPV16(+)] patients given the key roles of both HPV status and *miRNA* polymorphisms in SCCOP development and prognosis. The patients with *miRNA146*rs2910164 GG and Gemin3rs197388 CC genotypes had a significantly better DFS than those with the corresponding CG+CC (*P*_Log-rank_ = 0.048) and CA+AA genotypes (*P*_Log-rank_ = 0.048), respectively, while patients with *miRNA196*rs11614913 CC genotype had significantly worse DFS than those with CT+TT genotypes, respectively (*P*_Log-rank_ = 0.044) (Figure 1B). To control potential effects from other confounders, we adjusted analyses with other prognostic confounders. We found that *miRNA146*rs2910164 GG and *miRNA631*rs5745925 CC genotypes had 50% and 60% lower risks for recurrence than the corresponding CG+CC and CA+AA genotypes, respectively (aHR, 0.5; 95% CI, 0.3-1.0 and aHR, 0.4; 95% CI, 0.1-1.0, respectively) (Table 3). Moreover, the *miRNA196*rs11614913 CC genotype had an approximately 2 times significantly higher risk of disease recurrence than the *miRNA196*rs11614913 CT+TT genotypes (aHR, 2.1; 95% CI, 1.1-3.9).

**Table 3:** Association between *miRNA*-related genotypes and HPV-positive SCCOP recurrence in patients with SCCOP (N = 324)

Genotype	No. of rec. of patients/no. of patients	5-year rec. rate	Log-rank *P*	aHR*, 95% CI
*miRNA146rs2910164*				
GG (ref.)	14/146	0.10	0.048	0.5 (0.3-1.0)
CG+CC	31/178	0.20		1.0
*MiRNA196rs11614913*				
CC (ref.)	16/76	0.21	0.044	2.1 (1.1-3.9)
CT+TT	29/248	0.13		1.0
*MiRNA149* rs2292832				
CC (ref.)	22/159	0.17	0.896	0.8 (0.5-1.5)
CT+TT	23/165	0.18		1.0
*MiRNA499* rs3746444				
TT (ref.)	6/32	0.21	0.846	1.3 (0.5-3.2)
CT+CC	39/292	0.18		1.0
*MiRNA492rs2289030*				
CC(ref.)	37/258	0.16	0.841	1.0 (0.5-2.2)
CG+GG	8/66	0.15		1.0
*MiRNA423rs6505162*				
TT(ref.)	19/174	0.12	0.127	0.7 (0.4-1.3)
CT+CC	26/150	0.18		1.0
*Gemin4rs910924*				
CC(ref.)	12/93	0.14	0.501	0.9 (0.5-1.8)
CT+TT	33/231	0.15		1.0
*Gemin3rs197388*				
CC(ref.)	2/33	0.06	0.048	0.4 (0.1-1.0)
CA+AA	43/291	0.16		1.0
*TRBPrs784567*				
CC(ref.)	16/122	0.16	0.793	1.1 (0.6-2.0)
CT+TT	29/202	0.14		1.0

HR, hazard ratio.

* Adjusted for age, sex, ethnicity, smoking status, alcohol use, stage, comorbidity, and treatment.

† Reference group.

**Figure 2A-B:**
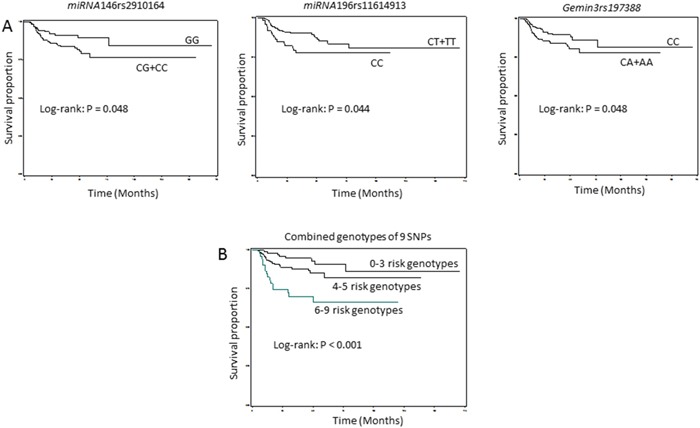
Kaplan-Meier estimates of the cumulative recurrence rates in HPV-positive SCCOP patients by *miRNA*-related genotypes (N = 324)

To assess the combined effects on risk of recurrence, we combined risk genotypes of 9 SNPs of *pre-miRNAs*. The univariate survival analysis according to the number of combined risk genotypes; and the DFS was significantly different among different groups with different combined risk genotypes (*P_L_*_og-rank_ < 0.001) (Figure 2A). According to the risk associated with each of the 9 individual SNPs, we grouped patients into three new groups based on the numbers of risk genotypes of each polymorphism. After combination, a significant trend for increased recurrence risk with increasing number of risk genotypes was observed (*P_trend_* < 0.001) (Table 4). For example, compared with patients carrying 0-3 risk genotypes, those carrying greater than 6 risk genotypes had 3-fold increased risk of recurrence (aHR, 3.0; 95% CI, 1.7-4.2).

**Table 4:** Association between combined *miRNA*-related genotypes and SCCOP recurrence in patients with SCCOP (N = 1008)

Combined Genotype	No. of rec. of patients/no. of patients	5-year rec. rate		Log-rank *P*	aHR*, 95% CI
*0-3*	47/376	0.12		< 0.001	1.0
4-5	97/508	0.20			1.7 (1.2-2.4)
6-9	37/124	0.31			3.0 (1.7-4.2)
*P_trend_*			< 0.001		

HR, hazard ratio.

* Adjusted for age, sex, ethnicity, smoking status, alcohol use, stage, comorbidity, and treatment.

†Reference group.

Given the roles of human papillomavirus (HPV) in development of SCCOP, we further assessed the associations between the combined SNPs and recurrence risk of tumor HPV16/18-positive SCCOP patients. Among 324 HPV16/18-positive SCCOP patients, the groups with different combined risk genotypes had significant differences in DFS (*P*_Log-rank_ < 0.001) (Figure 2B). Furthermore, the effect estimates of combined risk genotypes on risk of recurrence were statistically significant (*P*_Trend_ < 0.001) as shown in Table 5. As indicated, patients with greater than 6 risk genotypes had 5-fold increased risk of recurrence than those with 0-3 risk genotypes (aHR, 5.0; 95% CI, 2.1-12.0).

**Table 5:** Association between combined *miRNA*-related genotypes and HPV-positive SCCOP recurrence in patients with SCCOP (N = 324)

Combined Genotype	No. of rec. of patients/no. of patients	5-year rec. rate		Log-rank *P*	aHR*, 95% CI
*0-3*	8/106	0.07		< 0.001	1.0
4-5	21/165	0.19			2.2 (1.0-5.0)
6-9	16/53	0.34			5.0 (2.1-12.0)
*P_trend_*			0.0002		

HR, hazard ratio.

* Adjusted for age, sex, ethnicity, smoking status, alcohol use, stage, comorbidity, and treatment.

†Reference group.

## DISCUSSION

From this study, we observed significant effects on risk of SCCOP recurrence for certain individual or the combined risk genotypes of the 9 polymorphisms, particularly for those whose tumors were HPV16/18-positive. These results indicate that *miRNA*-related polymorphisms may individually or jointly serve as susceptibility markers for recurrence of SCCOP patients, particularly HPV16/18-positive SCCOP patients.

Previous studies has shown that *in vivo* and *in vitro* overexpression of some *miRNAs* may affect cancer progression and poor outcomes [13, 20], while downexpression of other miRNAs may modulate sensitivity to ionizing radiation [21]. It is likely that *miRNAs* expression patterns could be used as clinically useful predictors for outcome of SCCOP [20]. *MiRNA* variants might cause changes of *miRNA* expression levels and person to person variations in several important systems (e.g., inflammatory/immune, apoptosis). These *miRNA* variants could modulate the immune/inflammatory responses and apoptotic capacity, eventually leading to different viral immune escape and evasion of apoptosis. Thus, these genetic variations can cause different clinical outcomes among SCCOP patients after radiotherapy.

*miRNAs* play important roles in apoptosis and inflammation/immune responses [22]. Genetic variations in these molecular pathways might affect both HPV clearance and HPV escape of immune surveillance; thus resulting in different tumor HPV status and prognosis of SCCOP patients. Both *in vitro* and *in vivo* study reported that m*iRNAs* are involved in inflammation and innate and adaptive immune responses to stress [23], in which miRNAs may target key genes in the regulation of these responses to environmental insults including HPV infection [23]. Functional experiments further suggest that *miRNAs* are involved in both regulations of cytokine signaling and cell death pathways [24, 25]. Thus, *miRNA*-related variants could affect apoptotic capacity and subsequently sensitivity of response to radiotherapy.

Although the roles of these 9 *miRNAs*-related SNPs in cancer risk and prognosis have been previously investigated [16, 17, 26–29], to date none have evaluated associations of the 9 SNPs with recurrence of SCCOP patients. Previous studies have indicated that polymorphisms within either *pre-miRNAs* or *miRNA*-binding sites could likely influence expressions of the *miRNA* targets, resulting in different clinical outcome [26–28]. In a lung cancer study, *hsa-mir196a2* rs11614913 CC significantly caused a decreased survival [16]. Recently, *hsa-mir-*196a2 rs11614913 SNP was found to modulate survival of pharyngeal cancer [30]. Moreover, we also previously reported that *hsa-mir*-146a GG and *hsa-mir*-196a2 polymorphism significantly modulated survival of SCCOP patients, particularly in patients with HPV16-positive tumors [31]. After adjustment with other potential prognostic factors, the significant association of reduced risk of recurrence in SCCOP patients with this polymorphism remained significant [31].

Some studied also reported that *miRNA423* rs6505162 CG+CC genotypes had a significantly increased risk of recurrence than their corresponding GG genotype in colorectal cancer [32] but not for gastric cancer [33]. Yu et al reported that the patients carrying the *miRNA492*rs2289030 CG+GG genotypes had significantly reduced risk of survival compared to those with CC genotype in hepatocellular carcinoma [34]. However, such significant associations were not found for both *miRNA423* rs6505162 and *miRNA492*rs2289030 in lung cancer [35]. More recently, three studies have reported the associations of *Gemin4*rs910924 and *TRBP*rs78567 polymorphisms with cancer prognosis [36–38] and only *TRBP* CT+TT genotypes had increased risk of recurrence in Hodgkin lymphoma patients. Unfortunately, such associations were not observed in this current study. Few studies on associations of Gemin3rs197388 with cancer recurrence have been previously reported, while in this study patients with Gemin3rs197388 CC genotype had a significantly lower risk than the patients carrying CA+AA genotypes, particularly in tumor HPV16-positive SCCOP.

Since it is biologically likely that the risk-conferring effect of each of individual polymorphisms is only minimal, to more powerfully explore the effects of *miRNA*-related polymorphisms on recurrence risk, we may collapse all the risk genotypes to assess their combined effects on recurrence risk. Through such an approach, we may identify a trend toward an increasing SCCOP recurrence risk with an increasing number of risk genotypes that are in a dose-dependent manner. However, in this study no any pronounced associations of each of *miRNA*-related polymorphisms with risk were found, but a combined effect of these polymorphisms on recurrence of SCCOP patients was noticed, with a significant risk trend in a dose-response manner. Such effects on recurrence risk was particularly high in patients with HPV16-positive tumors. This finding implies that the simultaneous evaluation of these miRNA-related polymorphisms more likely identify risk markers of cancer recurrence than variation at a single locus. A combined analysis of multiple factors thus may powerfully characterize high-risk patients.

We selected SNPs in miRNA-related genes using a broader and pathway-based approach rather than a narrow candidate-SNP approach, through which we can assess the combined effects of a panel of polymorphisms which act in the miRNA-related pathways. Such analysis may result in amplified associations of these polymorphisms with the recurrence risk among SCCOP patients. The putatively functional polymorphisms in miRNA processing pathway could influence expression or biologically functional alterations of their host genes and thus play a role in miRNA-mediated cancer prognosis. Furthermore, miRNAs have a complex and overlapping range of activities, which will enable us to hardly isolate individual polymorphisms. Genetic variation of miRNAs may alter miRNA expression or biological function, disrupt the networks of miRNAs in various cellular pathways, and thus affect cancer risk and outcome.

How these *miRNA*-related variants exactly influence recurrence of SCCOP is still unknown, particularly for tumor HPV16-positive patients, we speculate that it is likely that these SNPs might have their own biological functions or are in linkage disequilibrium with other functional polymorphisms of *miRNAs*, leading to functional changes or altered regulations of the miRNAs. The resulted changes in miRNAs could cause individual variations in sensitivity to radiotherapy. Although we found some significant associations between *miRNA*-related polymorphisms and recurrence risk among SCCOP patients, larger studies are warranted to validate our results due to several inherent limitations in the present study including other potential confounders, selection bias, misclassification of HPV16 tumor status, multiple ethnicities inclusion, short duration of follow-up, and a hospital-based nature of the study. Furthermore, data on smoking duration and intensity, such as pack-year, should be included for adjustment and similar analysis with large sample size of HPV16-negative SCCOP patients should be considered in our future studies. In conclusion, genetic variants of *pre-miRNAs* may modulate the risk of recurrence of SCCOP patients, particularly in HPV16-positve SCCOP tumors.

## PATIENTS AND METHODS

### Study subjects

Patients with SCCOP were consecutively enrolled from May 1995 through April 2010, as described previously [22]. All patients had newly diagnosed, histopathologically confirmed, untreated SCCOP; patients of all ages, sexes, ethnicities, and clinical stages were recruited. Patients with distant metastases at presentation were excluded. Approximately 95% of contacted patients consented to enrollment in the study. All subjects signed an informed consent form that had been approved by the institutional review board of The University of Texas MD Anderson Cancer Center (Houston, Texas). The more details of patients' follow-up and clinical data were previously described [39, 40]. In this study, we clearly defined and distinguished the recurrence from second primary tumors to minimize the over- or underestimation of associations. The sixth edition of the American Joint Committee on Cancer TNM staging system was used to determine disease stage at the time of presentation for study patients. Definitive radiotherapy was defined if the patients received radiation treatment only or radiation treatment in combination with any other therapeutic modalities. Medical comorbidities were classified according to a modification of the Kaplan-Feinstein comorbidity index (Adult Comorbidity Evaluation 27).

### Genotyping

From each blood sample, a leukocyte pellet was obtained from the buffy coat by centrifugation of 1 mL of whole blood. The pellet was used for genomic DNA extraction with the DNA Blood Mini Kit (Qiagen Inc., Valencia, CA) according to the manufacturer's instructions. A polymerase chain reaction–restriction fragment-length polymorphism (PCR-RFLP) assay was used to genotype the polymorphisms of interest. The miRNA polymorphisms, restriction enzymes, and primers used for each SNP are listed in Table 6. All the restriction enzymes used were from New England Biolabs, Inc. (Beverly, MA). Approximately 10% of samples were retested, demonstrating 100% concordance.

**Table 6:** Genotyping assays of *MicroRNA*-related SNPs

*MicroRNA*-related SNPs	SNP (Base Change)	Primers Used (Sense/Antisense)	PCR Product (bp)	Enzymes	Digested Products
*miR146* rs2910164	G > C	5′-AGGAGGGGTCTTTGC ACCATC-3′ 5′-CCCAGCTGA AGA ACTGAA CTGCA-3′	113	*PstI*	G allele: 113 bp C allele: 89/24 bp
*miR149* rs2292832	C > T	5′-ATGTCCAGGACCACA ACCTGT-3′ 5′-CACCTCTCACACCCCCTCAC-3′	337	*PvuII*	C allele: 337 bp T allele: 220/117 bp
*miR196* rs11614913	C > T	5′-CAGCTGATCTGTGGCTTAGGT-3′ 5′-GAAAACCGACTGATGTAACCCAG-3	93	*MvaI*	T allele: 93 bp C allele: 70/23 bp
*miR499* rs3746444	C > T	5′-ACGGGAAGCAGCACAGACTTG-3′ 5′-TGTTTAACTCCTCTCCACGTGTAC-3′	52	*BsrGI*	C allele: 52 bp T allele: 27/25 bp
*miR492 rs2289030*	G > C	5′-CCAAACTTGGTTCAGAAGTCAAC-3′ 5′-CAACAATGCTCAACTGGCTGCTC-3′	184	PstI	G allele:184 bp C allele: 161/23bp
*MiR423 rs6505162*	C > T	5′-GGCCCCTCAGTCTTGCTTCCTTC-3′ 5′-GGGGAGAAACTCAAGCGCGGAT-3′	209	*HincII*	T allele: 209 bp C allele: 128/81 bp
*Gemin4 rs910924*	T > C	5′-GCTCCAGAGGGCACACGAGCATC-3′ 5′-CTCAGCAATGGACTTTAAGAAGGA-3′	251	HaeII	C allele: 251bp T allele:164/87 bp
*Gemin3 rs197388*	A > C	5′-CTGAATCTACGCCTGTGGATGAAC-3′ 5′-TGGTGGTTGTTCCAAAGAAATAGT-3′	185	BsiEI	A allele: 185 bp C allele: 132/53 bp
*TRBP rs784567*	T > C	5′-TTACCTAGACGCCGGAGGGGATC-3′ 5′-GAGCCCTGCGGAAACAGAGATGA-3′	199	AvaII	C allele: 199 bp T allele: 125/74 bp

### Determination of tumor HPV16 status

Paraffin-embedded tissue biopsies or specimens from study patients with tissues available were used to extract DNA for tumor HPV16 detection using PCR and in situ hybridization methods, described previously [41]. For quality control, a subset of samples was re-assayed for tumor HPV16 status. The results of the re-run samples were 100% concordant with the original results.

### Statistical analysis

The primary endpoint of the study was recurrence. The time to event was calculated from the date of diagnosis of the index SCCOP to the date of clinically detectable recurrence (local, regional, or distant). Patients who were not known to have had an event at the date of last contact and patients who were lost to follow-up or died of other or unknown causes were censored. We first used Student's *t* test to compare the mean age and follow-up time between patients with and without recurrence. The associations between individual epidemiologic risk factors, clinical characteristics (including stage, comorbidity, and treatment variables), and time to recurrence were initially assessed using univariate Cox proportional hazards regression models. An examination of Kaplan-Meier survival curves and log-minus-log survival plots indicated that the data were consistent with the assumptions of the Cox proportional hazard regression models. The associations between variables and disease-free survival (DFS) were evaluated using the log-rank test. We assessed the associations between individual epidemiologic risk factors, clinical characteristics (including stage, comorbidity, and treatment variables), and time to recurrence using both univariate and multivariate Cox proportional hazards regression models. Associations between genotypes and risk of recurrence were quantified by calculating the hazard ratios (HRs) and their 95% CIs. The Cox model included adjustment for potential confounders, including age, sex, ethnicity, smoking status, alcohol use status, tumor stage, comorbidity, and treatment. For all analyses, statistical significance was set at *P* < 0.05, and all tests were two-sided. SAS software (version 9.2.3; SAS Institute) was used to perform all statistical analyses.

## Abbreviations

miRNA: microRNA
SCCHN: squamous cell carcinoma of the head and neck
HR: hazard ratio
CI: confidence interval
PCR: polymerase chain reaction
SNPs: single nucleotide polymorphisms
HPV: human papillomavirus
SCCOP: squamous cell carcinomas of the oropharynx
